# Non-linear relationship between sleep duration and blood pressure in children with short stature

**DOI:** 10.3389/fped.2023.1302750

**Published:** 2023-11-06

**Authors:** Qianqian Zhao, Mingming He, Mei Zhang, Yuntian Chu, Bo Ban

**Affiliations:** ^1^ Department of Endocrinology, Affiliated Hospital of Jining Medical University, Jining Medical University, Jining, China; ^2^ Department of National Telemedicine Center of China, Chinese Research Center for Behavior Medicine in Growth and Development, Jining, China; ^3^ National Telemedicine Center of China, The First Affiliated Hospital of Zhengzhou University, Zhengzhou, China

**Keywords:** systolic blood pressure, diastolic blood pressure, sleep duration, cardiovascular disease, short stature

## Abstract

**Background:**

Evidence regarding the relationship between sleep duration and blood pressure is controversial. Therefore, the aim of this study was to investigate the relationship between sleep duration and blood pressure in children with short stature.

**Methods:**

A total of 1,085 participants with short stature were enrolled from the Affiliated Hospital of Jining Medical University in China. The variables involved in this study included sleep duration, anthropometric indicators and biochemical parameters. Sleep duration was evaluated in a face-to-face interview.

**Results:**

The average age of the 1,085 selected participants was 10.2 ± 3.5 years old, and approximately 763 (70.32%) of them were male. The results of adjusted linear regression showed that sleep duration was negatively associated with systolic blood pressure *z* scores (SBP-Z) and diastolic blood pressure *z* scores (DBP-Z) after adjusting for confounders (β −0.07, 95% CI −0.13, −0.01 *P* = 0.038; β −0.05, 95% CI −0.10, −0.01 *P* = 0.035, respectively). A nonlinear relationship was detected between sleep duration and blood pressure, including SBP-Z, DBP-Z and mean arterial pressure *z* scores (MAP-Z). The inflection point of the nonlinear relationship between sleep duration and SBP-Z is 10 h, and the inflection point of DBP-Z and MAP-Z is 8 h.

**Conclusion:**

This study revealed a nonlinear relationship between sleep duration and blood pressure in children with short stature. The findings suggest that the optimal sleep duration in children with short stature was 8–10 h, and sleep durations either too short or too long were associated with increased blood pressure levels.

## Introduction

Childhood hypertension is considered an important risk factor for advanced cardiovascular disease (CVD) (1). There is growing evidence that high blood pressure continues from childhood into adulthood, which indicates that children with high blood pressure are more likely to suffer from hypertension later in life (2). Considering that there is not a single cut-off point to define high blood pressure in children and adolescents (3), it is more difficult for young people to be recognized as having high blood pressure compared to adults. A growing number of studies have given considerable attention to screening for elevated blood pressure and have confirmed relevant factors in youths, especially in obese children, who are prone to having unfavourable blood pressure levels (4–6). However, there is increasing evidence that short stature is also a risk factor for CVD, and recent studies have reported that an inverse relationship between height and blood pressure is also exists in children (7, 8). Therefore, these studies suggest the need to analyse the blood pressure status and the associated factors, especially in children and adolescents with short stature.

Previous studies have demonstrated that elevated blood pressure as assessed in childhood is a predictor of increased risk of CVD in adults (9, 10). The assessment of childhood CVD risk based on elevated blood pressure may be useful for preventing CVD in adulthood (11). Previous studies have evaluated numerous factors that influence blood pressure levels, and major risk factors for hypertension have been identified, including age, body mass index (BMI), dyslipidaemia, diet and lifestyle habits (12); however, sleep is an often overlooked factor. Sleep plays an important role in the daily life of adults and the growth of children and adolescents, and adequate sleep is essential for everyday functioning and health. However, sleep deprivation is very common, with up to 30% of people affected by sleep deprivation during childhood and adolescence (13). Sleep deprivation in children increases the likelihood of poor behaviour and physical health consequences. In particular, an association between short sleep duration and an increased risk of cardiovascular events has been well established (14). Recent studies have suggested that the effect of sleep duration on CVD may be related to elevated blood pressure (15). Studies on the relationship between sleep duration and blood pressure have gradually increased (16). However, findings from previous studies regarding the relationship between sleep duration and blood pressure have been controversial in both children and adults (17–26), and the relationship between sleep duration and blood pressure has been found to be U-shaped (26), negatively correlated (19) or unrelated (20, 26). However, evidence of a relationship between sleep duration and blood pressure is lacking in children with short stature. Therefore, the aim of this study was to investigate the relationship between sleep duration and blood pressure in Chinese children with short stature.

## Methods

### Study population

The present study is a secondary analysis of our prospective cohort study. All the subjects enrolled were in the GDDSD study (http://www.chictr.org.cn, ChiCTR1900026510), an ongoing prospective, observational, open cohort study that is evaluating the aetiology of growth and development diseases and the long-term safety and effectiveness of growth hormone therapy in a real-life clinical setting (27). A total of 1,085 children and adolescents with short stature (763 males and 322 females) from the Affiliated Hospital of Jining Medical University between March 2013 and October 2020 were recruited. The average age of the participants was 10.2 ± 3.5 years. Subjects were eligible if their height standard deviation scores (SDS) were more than 2 SDS lower than the average of individuals of the same race, age and sex. The exclusion criteria were as follows: (1) chronic diseases; (2) skeletal dysplasia; (3) chromosomal abnormalities, including Noonan syndrome and Turner syndrome; and (4) missing data on sleep duration and blood pressure.

### Anthropometric measurement

Height and weight were measured using a standard procedure with the participants wearing no shoes or coats. Height was measured using a stadiometer (Nantong Best Industrial Co., Ltd., Jiangsu, China), which is accurate to 0.1 cm. An electronic scale (Wuxi Weigher Factory Co., Ltd., Jiangsu, China) was used to measure weight to the nearest 0.1 kg. Body mass index (BMI) was calculated as weight (kg)/height squared (m^2^). The SDS of height and BMI were calculated based on the growth charts of normal Chinese children (28, 29). Blood pressure measurements were performed after a 10-min rest with the children in a seated position, and the blood pressure was measured three times on the right arm with an electronic sphygmomanometer (Omron HBP-1300, Dalian, China). The average of the three measurements was used in the analyses. Systolic blood pressure (SBP) and diastolic blood pressure (DBP) were also expressed in SBP *z* scores (SBP-Z) and DBP *z* scores (DBP-Z) related to age, gender and height for every individual (30). The mean arterial pressure (MAP) was calculated as DBP plus one-third of the pulse pressure. Pubertal maturity was assessed by a trained physician who performed a physical examination and assigned each child a maturity level according to the Tanner stage (31). Boys with testicular volume less than 4 ml and no pubic hair and girls with undeveloped breasts and no pubic hair were considered prepubescent. Sleep duration was determined by parental report or self-report.

### Laboratory measurements

All participants fasted for at least eight hours before blood samples were taken for biochemical measurements. The serum insulin-like growth factor-1 (IGF-1) concentration was measured by chemiluminescence assay (DPC IMMULITE 1,000 analyser, Siemens, Germany), and the intra- and inter-assay coefficients of variation (CVs) were 3.0% and 6.2%, respectively. Fasting plasma glucose (FPG), triglycerides (TG), total cholesterol (TC), high-density lipoprotein-cholesterol (HDL-C), and low-density lipoprotein-cholesterol (LDL-C) were analysed by an autobiochemical analyser (Cobas c702, Roche; Shanghai, China). The IGF-1 SDS was calculated using the reference values of healthy children of the same age and sex (32).

### Statistical analysis

Continuous variables are expressed as the mean ± standard deviation or median (interquartile range), while categorical variables are presented as numbers and percentages. Univariate analysis was performed to estimate the association between SBP-Z and sleep duration as well as the other variables. Smooth curve fitting was applied to explore nonlinear associations between sleep duration and SBP-Z. In addition, smooth curve fitting was also used to analyse the relationship between sleep duration and DBP-Z, MAP *z* scores (MAP-Z). Furthermore, to examine the independent association of sleep duration and SBP-Z, we used multivariate linear regression and two piecewise linear regressions. A two-sided *P* < 0.05 was considered statistically significant. Data were analysed using R 3.6.1 (https://www.R-project.org) and EmpowerStats (https://www.Empowerstats.com, X&Y Solutions, Inc.; Boston, MA).

## Results

### Baseline characteristics of participants

Table 1 shows the descriptive characteristics of the study population at baseline. A total of 1,085 individuals participated in the study. The average age of the children was 10.2 ± 3.5 years old, and 763 (70.32%) of them were boys. The mean height SDS of the participants was −2.70 ± 0.63. Among the study population, 737 participants were prepubescent, accounting for 67.93% of the sample. The assessment of sleep habits showed that the average sleep duration per night in the population study sample was 9.20 ± 0.96 h. The mean SBP-Z and SBP level were 0.73 (0.08–1.45) and 105.71 ± 11.98 mmHg, respectively. The mean DBP-Z and DBP level were 0.35 (−0.07–0.84) and 62.22 ± 8.72 mmHg, respectively.

**Table 1:** Clinical and biochemical characteristics.

Variables	All
Number	1,085
Sex (male %)	763 (70.32%)
Age (years)	10.2 ± 3.5
Height (cm)	125.43 ± 17.94
Height SDS	−2.70 ± 0.63
Body weight (kg)	27.72 ± 11.49
BMI (kg/m^2^)	18.01 ± 3.47
BMI SDS	0.12 (−0.69–1.03)
Sleep duration (hours)	9.20 ± 0.96
IGF-1 (ng/ml)	168.00 (98.25–252.75)
IGF-1 SDS	−0.96 (−1.74–0.11)
SBP (mmHg)	105.71 ± 11.98
SBP-Z	0.73 (0.08–1.45)
DBP (mmHg)	62.22 ± 8.72
DBP-Z	0.35 (−0.07–0.84)
MAP (mmHg)	76.71 ± 8.60
MAP-Z	0.50 (0.07–0.95)
FPG (mmol/L)	4.86 ± 2.11
TG (mmol/L)	0.65 (0.51–0.86)
TC (mmol/L)	3.86 ± 0.73
HDL-C (mmol/L)	1.45 ± 0.30
LDL-C (mmol/L)	2.11 ± 0.59
Pubertal stage	
In prepuberty (%)	737 (67.93%)
In puberty (%)	348 (32.07%)

Height SDS, height standard deviation scores; BMI SDS, body mass index standard deviation scores; IGF-1 SDS, insulin like growth factor-1 standard deviation scores; SBP-Z, systolic blood pressure *z* scores; DBP-Z, diastolic blood pressure *z* scores; MAP-Z, mean arterial pressure *z* scores; FPG, fasting plasma glucose; TG, triglyceride; TC, total cholesterol; HDL-C, high density lipoprotein-cholesterol; LDL-C, low density lipoprotein cholesterol. Continuous variables are presented as the mean ± standard deviation or median (interquartile range). Categorical variables are displayed as number (percentage).

### Variables associated with blood pressure in the participants

The analysis results of the relationship between blood pressure and clinical parameters are provided in Table 2. In the univariate linear regression analysis, we observed a significant negative association between sleep duration and SBP-Z and DBP-Z (both *P* < 0.05). In addition, the relationship between age, sex and SBP-Z and DBP-Z remained negative, while other variables, including height SDS, body weight, IGF-1 SDS, TG, TC and LDL-C, were positively associated with SBP-Z (all *P* < 0.05). However, there were no significant associations between SBP-Z and pubertal stage, BMI SDS, FPG or HDL-C (all *P* > 0.05).

**Table 2:** Association between blood pressure and different variables.

Variables	SBP-Z			DBP-Z		
	β	(95% CI)	*P*-value	β	(95% CI)	*P*-value
Age (years)	−0.02	(−0.03, −0.01)	0.041	−0.03	(−0.04, −0.01)	<0.001
Height SDS	0.13	(0.05, 0.21)	0.002	0.15	(0.09, 0.22)	<0.001
Body weight (kg)	0.01	(0.01, 0.01)	0.001	0.01	(0.01, 0.01)	0.003
BMI SDS	0.04	(−0.01, 0.09)	0.081	0.03	(−0.01, 0.07)	0.144
Sleep duration (hours)	−0.07	(−0.13, −0.01)	0.034	−0.03	(−0.08, −0.01)	0.048
IGF-1 SDS	0.11	(0.07, 0.16)	<0.001	0.06	(0.02, 0.10)	0.001
FPG (mmol/L)	−0.01	(−0.04, 0.02)	0.394	−0.01	(−0.03, 0.02)	0.721
TG (mmol/L)	0.19	(0.05, 0.34)	0.010	0.01	(−0.11, 0.12)	0.936
TC (mmol/L)	0.09	(0.01, 0.18)	0.024	0.03	(−0.04, 0.09)	0.436
HDL-C (mmol/L)	−0.01	(−0.02, 0.01)	0.402	−0.01	(−0.01, 0.01)	0.803
LDL-C (mmol/L)	0.16	(0.06, 0.26)	0.002	0.03	(−0.05, 0.11)	0.450
Sex						
Male	Reference			Reference		
Female	−0.28	−0.28 (−0.41, −0.15)	< 0.001	−0.17	(−0.27, −0.06)	0.002
Pubertal stage						
In prepuberty (%)	Reference			Reference		
In puberty (%)	−0.08	−0.08 (−0.20, 0.05)	0.224	−0.13	(−0.23, −0.03)	0.009

Height SDS, height standard deviation scores; BMI SDS, body mass index standard deviation scores; IGF-1 SDS, insulin like growth factor-1 standard deviation scores; SBP-Z, systolic blood pressure *z* scores; DBP-Z, diastolic blood pressure *z* scores; FPG, fasting plasma glucose; TG, triglyceride; TC, total cholesterol; HDL-C, high density lipoprotein-cholesterol; LDL-C, low density lipoprotein cholesterol. *P* < 0.05 is considered to be statistically significant.

### Independent association between sleep duration and blood pressure

**Figure 1:**
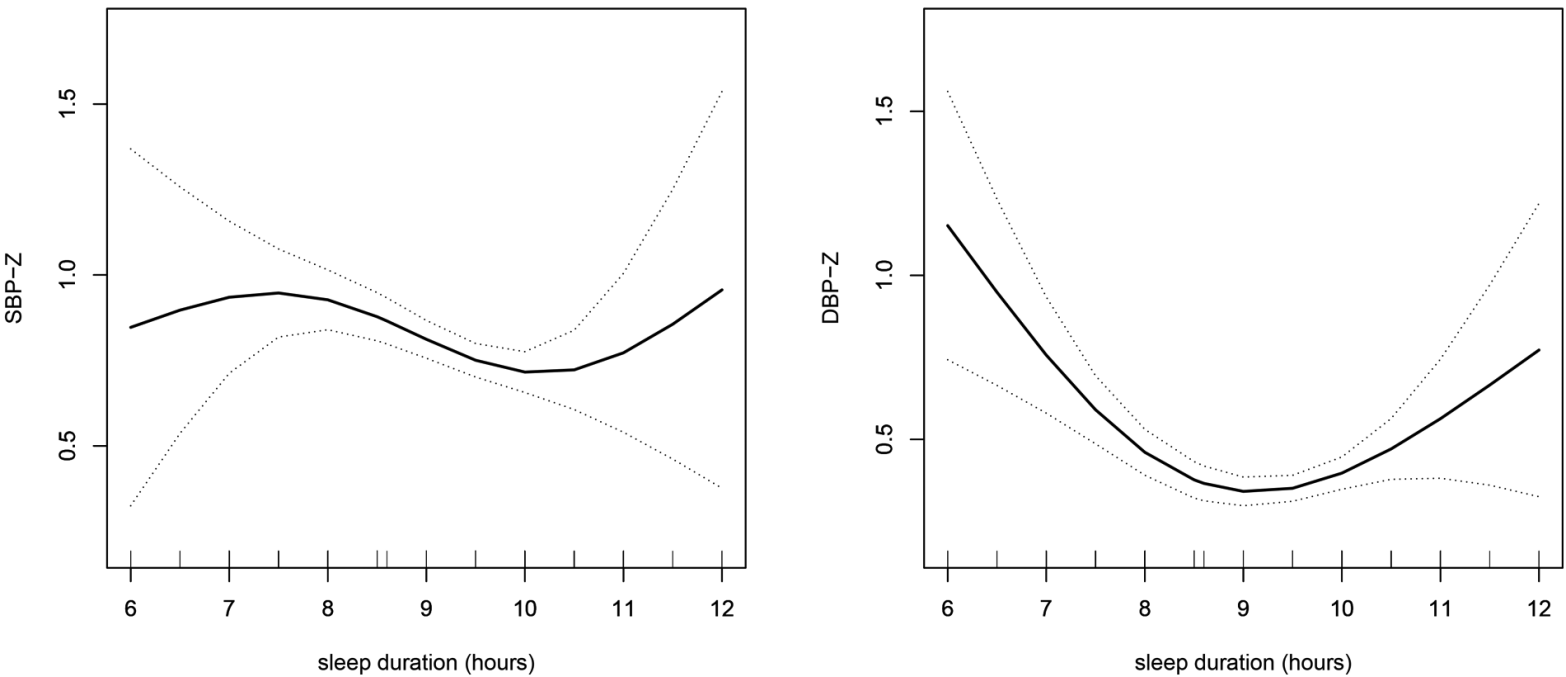
The relationship between sleep duration and blood pressure was determined by smooth curve fitting. Adjustment variables: age, sex, height, weight, IGF-1, TG and TC. SBP-Z, systolic blood pressure *z* scores; DBP-Z, diastolic blood pressure *z* scores; IGF-1, insulin like growth factor-1; TG, triglyceride; TC, total cholesterol.

**Figure 2:**
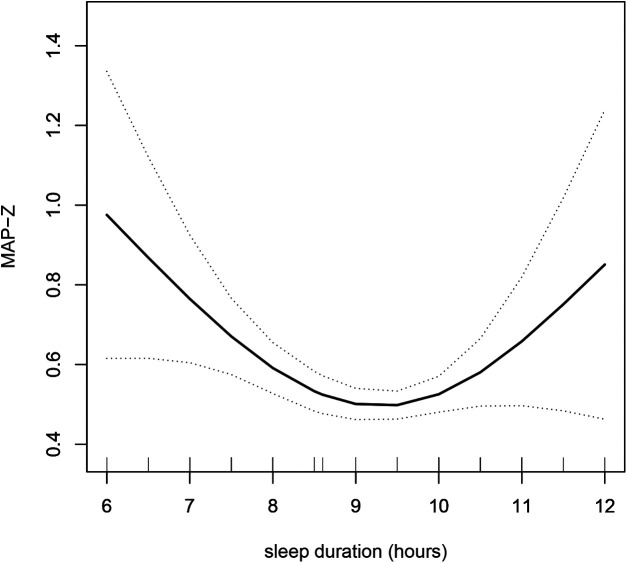
The relationship between sleep duration and MAP-Z was determined by smooth curve fitting. Adjustment variables: age, sex, height, weight, IGF-1, TG and TC. MAP-Z, mean arterial pressure *z* scores; IGF-1, insulin like growth factor-1; TG, triglyceride; TC, total cholesterol.

To explore whether there was a nonlinear relationship between sleep duration and blood pressure, smooth curve fitting was performed. The results showed that there was a nonlinear relationship between sleep duration and blood pressure (SBP-Z, DBP-Z and MAP-Z) after adjusting for potential confounding factors, and there was an inflection point (Figures 1, 2). Before the inflection point, sleep duration and blood pressure were negatively associated, and after the inflection point, sleep duration and blood pressure were positively associated. To investigate this finding further, we conducted linear regression and two piecewise linear regressions.

As presented in Table 3, it was observed through linear regression that after adjusting for confounding variables, sleep duration was independently negatively associated with SBP-Z (β −0.07, 95% CI −0.13, −0.01; *P* = 0.038). Moreover, in the two piecewise regressions, the results showed that the inflection point of sleep duration was 10 h. If sleep duration was less than 10 h, there was a negative association between sleep duration and SBP-Z (β −0.10, 95% CI −0.17, −0.03; *P* = 0.004), while if the sleep duration was greater than 10 h, there was a positive association between sleep duration and SBP-Z (β 0.34, 95% CI 0.04, 0.64; *P* = 0.025). In addition, we also analyzed DBP-Z, whose inflection point is 8 h, which is consistent with the relationship between SBP-Z and sleep duration.

**Table 3:** Threshold effect analysis for the relationship between the sleep duration and blood pressure.

Models	SBP-Z		DBP-Z	
	Adjusted β (95% CI)	*P*-value	Adjusted β (95% CI)	*P*-value
Model I				
One line slope	−0.07 (−0.13, −0.01)	0.038	−0.05 (−0.10, −0.01)	0.035
Model II				
Turning point	10		8	
<10 slope 1	−0.10 (−0.17, −0.03)	0.004	−0.61 (−0.83, −0.39)	<0.001
>10 slope 2	0.34 (0.04, 0.64)	0.025	0.02 (0.04, 0.01)	0.048
LRT test	0.006		<0.001	

Model I, linear analysis; Model II, non-linear analysis. LRT test, Logarithmic likelihood ratio test. (*p*-value < 0.05 means Model II is significantly different from Model I, which indicates a non-linear relationship); Adjustment variables: age, sex, height, weight, IGF-1, TG and TC. SBP-Z, systolic blood pressure *z* scores; DBP-Z, diastolic blood pressure *z* scores; IGF-1, insulin like growth factor-1; TG, triglyceride; TC, total cholesterol. *P* < 0.05 is considered to be statistically significant.

Furthermore, we analyzed the relationship between sleep duration and mean arterial pressure. Sleep duration was independently negatively associated with MAP-Z (β −0.05, 95% CI −0.10, −0.01; *P* = 0.021). Moreover, in the two piecewise regressions, the results showed that the inflection point of sleep duration was 8 h. If sleep duration was less than 8 h, there was a negative association between sleep duration and MAP-Z (β −0.43, 95% CI −0.64, −0.22; *P* < 0.001), while if the sleep duration was greater than 8 h, there was a positive association between sleep duration and MAP-Z (β 0.03, 95% CI 0.06, 0.01; *P* = 0.046) (Table 4).

**Table 4:** Threshold effect analysis for the relationship between the sleep duration and MAP-Z.

Models	MAP-Z	
	Adjusted β (95%CI)	*P*-value
Model I		
One line slope	−0.05 (−0.10, −0.01)	0.021
Model II		
Turning point	8	
<10 slope 1	−0.43 (−0.64, −0.22)	<0.001
>10 slope 2	0.03 (0.06, 0.01)	0.046
LRT test	<0.001	

Model I, linear analysis; Model II, non-linear analysis. LRT test, Logarithmic likelihood ratio test. (*p*-value < 0.05 means Model II is significantly different from Model I, which indicates a non-linear relationship); Adjustment variables: age, sex, height, weight, IGF-1, TG and TC. MAP-Z, mean arterial pressure *z* scores; IGF-1, insulin like growth factor-1; TG, triglyceride; TC, total cholesterol. *P* < 0.05 is considered to be statistically significant.

The log-likelihood ratio test was used to evaluate whether linear regression or two piecewise linear regressions could better represent the relationship between sleep duration and blood pressure. *P* < 0.05 indicated that piecewise linear regression could better reflect the true relationship between sleep duration and blood pressure. The results showed that *P* for the log-likelihood ratio test were all less than 0.05, so the two-piecewise linear regression used to fit the association between sleep duration and blood pressure could accurately represent the relationship (Tables 3, 4).

## Discussion

In this study, we observed a significant negative relationship between sleep duration and blood pressure in children with short stature. Furthermore, there was a nonlinear relationship between sleep duration and blood pressure. The optimal amount of sleep in children with short stature was found to be 8–10 h, and both short and long sleep durations were associated with increased blood pressure levels.

Over the past few years, many studies on sleep and blood pressure have been published, reporting conflicting results in both children and adults (17–26). A population-based cross-sectional study recruited 19,407 adults aged 18–79 years, and they observed that there were no significant associations between sleep duration and hypertension in the general population (20). Moreover, Morita N et al. conducted a study on 102 Japanese students with an average age of 11.9 ± 1.8 years and found that there was no relationship between sleep and blood pressure (33). In contrast, a meta-analysis of six prospective cohort studies examining the relationship between sleep duration and the risk of hypertension showed that short sleep duration was associated with an increased risk of hypertension (34). The results of our study are consistent with these studies. In this study, linear regression was used to find a negative association between sleep duration and blood pressure, and short sleep duration was related to high blood pressure levels.

Interestingly, smoothing curve fitting was applied, and it was found that there was a nonlinear relationship between sleep duration and blood pressure. We further performed piecewise linear regression to find the inflection point of 8 or 10 h. More specifically, when sleep duration was less than 8 or 10 h, there was a negative association between sleep duration and blood pressure, indicating that short sleep duration was related to a high blood pressure level. However, when sleep duration was more than 8 or 10 h, sleep duration was positively related to blood pressure. With increasing sleep duration, the blood pressure level increases significantly. This finding suggests that the optimal sleep duration in children with short stature was 8–10 h, and sleep durations either too short or too long can have a negative effect on blood pressure. This is consistent with the sleep duration recommended by the National Sleep Foundation (34). It is well established that shorter sleep duration is associated with elevated blood pressure levels (35); however, longer sleep duration was also associated with increased blood pressure. In the Sleep and Heart Health Study, an analysis of data from 5,910 participants aged 40 to 100 years (2,813 men and 3,097 women) found that sleeping nine hours or more was associated with a 30% increased risk of high blood pressure compared with sleeping seven to eight hours (36). In addition, consistent with our findings, Grandner M et al. assessed the relationship between sleep duration and blood pressure using combined data from the 2007–2017 National Health Interview Survey (NHIS) and 2013 Behavioural Risk Factor Surveillance System (BRFSS) from adults 18 years and older and reported that both short and long sleep durations are associated with increased hypertension risk (37).

Regarding the relationship between long sleep duration and blood pressure, the results of the study are also controversial (37–39). In addition to the reports that long sleep duration is related to high blood pressure (37), some studies have not found that long sleep duration is related to high blood pressure (38, 39). However, studies on the relationship between sleep duration and blood pressure in children with short stature are limited. This study reported that there is a nonlinear relationship between sleep duration and blood pressure, and sleep durations that are either too short or too long will increase blood pressure.

The underlying mechanisms by which sleep duration may contribute to increased blood pressure are complex and unclear. Some studies have suggested that short sleep duration may increase blood pressure by increasing sympathetic nervous system activity or by disrupting circadian rhythms and autonomic nervous responses (40, 41). In addition, short sleep periods may stimulate fatigue, irritability and stress, which increases the risk of elevated blood pressure (34). To date, no clear mechanism has been found to explain the relationship between long sleep duration and elevated blood pressure. However, some studies suggest that factors such as sleep disruption and depression may explain a link between long sleep and mortality (42, 43).

According to the hypertension guidelines, higher SBP and DBP are associated with an increased risk of CVD when they are considered separately. However, higher SBP is consistently associated with an increased risk of CVD after adjusting or stratifying DBP. In contrast, the association between DBP and CVD risk was not consistent after adjusting or stratifying SBP. In addition, mean arterial pressure can also predict the risk of CVD (44). Therefore, we analysed the relationship between sleep duration and blood pressure, including SBP-Z, DBP-Z and MAP-Z. The inflection point of the nonlinear relationship between sleep duration and SBP-Z is 10 h, and the inflection point of DBP-Z and MAP-Z is 8 h.

Several limitations of the present study are worth discussing. First, considering the cross-sectional nature of the data, it is impossible to establish a causal relationship between sleep duration and blood pressure. Second, sleep duration was based on subjective reports by parents or the participants. Although electroencephalogram (EEG) measurements of sleep duration are considered more accurate than subjective assessments, the results of subjective self-reporting correlate with those obtained by EEG methods (45). Finally, the population in the present study was composed of Chinese children and adolescents with short stature, and there was a nonlinear relationship between sleep duration and blood pressure. Different findings may be observed in other ethnic groups or disease populations, which is worthy of further study.

In conclusion, in the present study, a nonlinear relationship between sleep duration and blood pressure in children with short stature was observed. The findings suggest that the optimal sleep duration in children with short stature was 8–10 h, and sleep durations either too short or too long sleep durations were associated with increased blood pressure levels. Further studies are still needed to explore the potential biological mechanism of the association between sleep duration and blood pressure.

## Data Availability

The original contributions presented in the study are included in the article/Supplementary Material, further inquiries can be directed to the corresponding author.
